# Element Effects on High-Entropy Alloy Vacancy and Heterogeneous Lattice Distortion Subjected to Quasi-equilibrium Heating

**DOI:** 10.1038/s41598-019-51297-4

**Published:** 2019-10-15

**Authors:** E-Wen Huang, Hung-Sheng Chou, K. N. Tu, Wei-Song Hung, Tu-Ngoc Lam, Che-Wei Tsai, Ching-Yu Chiang, Bi-Hsuan Lin, An-Chou Yeh, Shan-Hsiu Chang, Yao-Jen Chang, Jun-Jie Yang, Xiao-Yun Li, Ching-Shun Ku, Ke An, Yuan-Wei Chang, Yu-Lun Jao

**Affiliations:** 1 Department of Materials Science and Engineering, National Chiao Tung University, 1001 University Road, Hsinchu, 30010 Taiwan; 2 Department of Materials and Optoelectronic Science, National Sun Yat-sen University, Kaohsiung, Taiwan; 3 Department of Materials Science and Engineering, University of California, Los Angeles, United States; 4 Graduate Institute of Applied Science and Technology, National Taiwan University of Science and Technology, Taipei, 10607 Taiwan; 5 R&D Center for Membrane Technology, Chung Yuan University, Taoyuan, 32023 Taiwan; 6 Department of Physics, College of Education, Can Tho University, Can Tho City, Vietnam; 7 Department of Materials Science and Engineering, National Tsing Hua University, Hsinchu, 30013 Taiwan; 8 National Synchrotron Radiation Research Center, Hsinchu, 30076 Taiwan; 9 Spallation Neutron Source, Oak Ridge National Laboratory, Oak Ridge, TN 37831 United States

**Keywords:** Metals and alloys, Mechanical properties

## Abstract

We applied Simmons–Balluffi methods, positron measurements, and neutron diffraction to estimate the vacancy of CoCrFeNi and CoCrFeMnNi high-entropy alloys (HEAs) using Cu as a benchmark. The corresponding formation enthalpies and associated entropies of the HEAs and Cu were calculated. The vacancy-dependent effective free volumes in both CoCrFeNi and CoCrFeMnNi alloys are greater than those in Cu, implying the easier formation of vacancies by lattice structure relaxation of HEAs at elevated temperatures. Spatially resolved synchrotron X-ray measurements revealed different characteristics of CoCrFeNi and CoCrFeMnNi HEAs subjected to quasi-equilibrium conditions at high temperatures. Element-dependent behavior revealed by X-ray fluorescence (XRF) mapping indicates the effect of Mn on the Cantor Alloy.

## Introduction

High-entropy alloys (HEAs) are emergent materials known for lattice distortion^1,2^ attributed to variations of atomic sizes^3^. The mechanical performance of HEAs is extraordinary^4,5^; however, the deformation mechanism of these alloys has not yet been conclusively determined^6–12^. Specifically, for high-temperature applications^13–15^, Lei *et al*.^16^ indicate that vacancies and interstices remain unclear for HEAs. Recently, Chen *et al*.’s first-principles calculations show higher-vacancy formation entropy for CoCrFeNi^17^. Elsayed *et al*.’s positron annihilation lifetime spectroscopy (PALS) results demonstrate that the point defects do not distribute uniformly in HEAs^18^. The thermodynamic analysis presented by Wang *et al*. concludes that the equilibrium vacancy concentrations and their clusters in HEAs substantially increase with the number of principle elements than those in pure metals and simple binary alloys^19^. However, Santodonato *et al*. showed element-dependent behavior, which is not solely attributable to configurational entropy effects^20^.

We applied Simmons–Balluffi methods^21^, Seeger’s approach^22^, and Bichile and Kulkarni’s thermal expansion formulation^23^ to quantify vacancy, as well as positron annihilation lifetime spectroscopy^24^ to characterize effective vacancy sizes. We carried out spatially resolved synchrotron X-ray diffraction and fluorescence mapping to investigate vacancy effects on the local structure and chemical distributions, respectively. Given the influence of Mn on the phase stability of the CrMn_x_FeCoNi HEAs as reported by Christofidou *et al*.^25^, we herein study CoCrFeNi and CoCrFeMnNi, using Cu as the benchmark reference to compare the two HEAs.

## Results and Discussion

### Thermal expansion of HEAs

The origin of thermal expansion in a pure metallic element is due to anharmonicity in its pair potential; however, the mechanism may be more complex in HEAs. Pamato *et al*. demonstrated that Debye heat capacity, anharmonicity of vibrational motion of atoms, electronic heat capacity, vacancy, and interstices can all influence thermal expansion^26^. Simmons and Balluffi^27^ explicitly formulated the difference between the dilation of the bulk specimen, $\Delta L/L$, and the change in the lattice parameter, $\Delta a/a$.

1 $$3(\Delta a/a)=p(T)+r(T)+x(T)$$

2 $$3(\Delta L/L)=q(T)+s(T)+y(T)$$

where *p*(*T*) and *q*(*T*) are the ideal thermal expansion without thermally generated defects, *x*(*T*) and *y*(*T*) are expansion arising directly from formation of defects, and *r*(*T*) and *s*(*T*) are the thermal expansion of the crystal due to the presence of lattice defects that alter the lattice frequency distribution and therefore internal energy. Certainly, more examinations that are rigorous are expected to elucidate thermal expansion evolution in HEAs.

**Figure 1:**
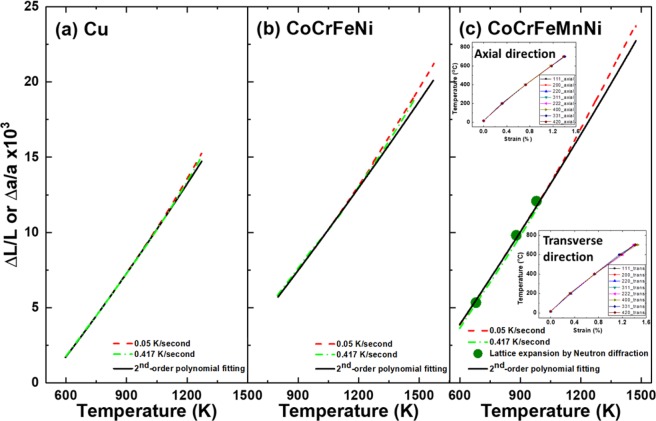
Measured strain *versus* temperature at the quasi-equilibrium and non-equilibrium states for (**a**) Cu, **(b**) CoCrFeNi, and (**c**) CoCrFeMnNi.

Figure 1 presents $\Delta L/L$ and $\Delta a/a$ of Cu, CoCrFeNi, and CoCrFeMnNi as a function of temperature. The bulk expansion $\Delta L/L$ subjected to quasi-equilibrium (red dashed line) and non-equilibrium (green dashed line) conditions were determined by using Netzsch dilatometry. The results for $\Delta a/a$ (solid black line) were calculated using second-order polynomial fitting following the methodology of Bichile and Kulkarni^23,28^, with a high-temperature X-ray diffractometer used to obtain a Grüneisen parameter for formulating the second-order polynomial fit for thermal expansion coefficient estimations^23^. It can be observed that *ΔL/L* increases with increasing temperature, and the second-order polynomial curve closely matches the results up to a temperature of approximately 1,000 K. At lower temperatures, both CoCrFeNi and CoCrFeMnNi HEAs have similar features as Cu, where non-equilibrium, quasi-equilibrium conditions, and the second-order polynomial curve all have the same expansion when subjected to heating, but deviate at temperatures higher than 1,000 K. Specifically, the quasi-equilibrium condition deviates from the second-order polynomial curve extrapolation at 900 K for CoCrFeNi and 1,000 K for CoCrFeMnNi.

We measured $\Delta a/a$ using a Vulcan neutron diffractometer for the *in situ* heating experiments and verified it by fitting a second-order polynomial curve for CoCrFeMnNi. Given the neutron interaction, a greater gauge volume yields multiple d spacings for different *hkl* planes from two orthogonal directions. In Fig. 1(c), the lattice thermal expansion of CoCrFeMnNi from room temperature up to 973 K (green circles) agrees with the second-order polynomial fitted curve and Laplanche *et al*.’s^29^ bulk measurements. With spallation neutron diffraction, the multi-lattice planes of (*1 1 1*), (*2 0 0*), (*2 2 0*), (3 *1 1*), (*2 2 2*), (*4 0 0*), (3 3 *1*), and (*4 2 0*) are illuminated simultaneously. The insets of Fig. 1(c) show that the lattice thermal expansion of CoCrFeMnNi is isotropic for various (*h k l*) values in both the axial and transverse orientations.

### Vacancy measurements

On the basis of Darken’s analysis of the Kirkendall effect and Simmons–Balluffi measurement of equilibrium vacancy concentration in face-centered cubic (FCC) metals, the vacancy diffusion mechanism in FCC metals and alloys has been widely accepted. Our earlier investigation^30^ and the experimental measurements reported here suggest that atomic diffusion in both CoCrFeNi and CoCrFeMnNi HEAs occurs by the vacancy diffusion mechanism. Comparing the thermal expansion results between the second-order polynomial extrapolation^23^ and the quasi-equilibrium thermal expansion following Seeger’s method^22^, we can quantify the vacancy concentration *X*_*V*_ for a metal system subjected to heating using Simmons and Balluffi’s classic formulation^31^:

3 $${X}_{V}=3(\frac{\varDelta L}{L}-\frac{\varDelta a}{a})$$

**Figure 2:**
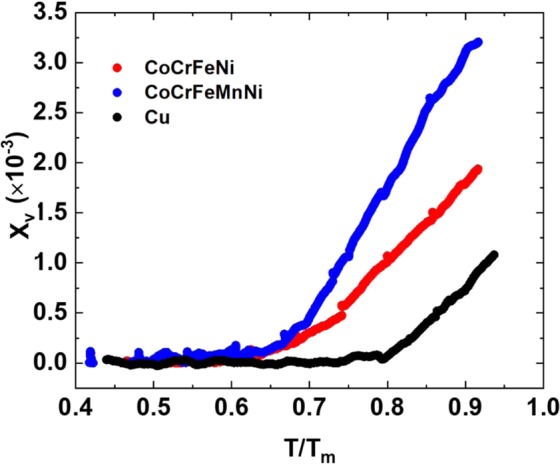
*X*_*v*_–homologous temperature (*T/T*_*m*_) curves of Cu, CoCrFeNi, and CoCrFeMnNi at the quasi-equilibrium state.

We follow Pamato *et al*.’s methodology^26^, presenting the evolution of vacancy formation in the alloys subjected to heating in Fig. 2. Here we demonstrate that the vacancies increase with the homologous temperature (*T/T*_*m*_) of each metallic system, where *T*_*m*_ is the melting temperature of materials. For the three alloys, the vacancies increase rapidly when the environmental temperatures are higher than 1,000 K. The observable onset temperature of vacancy formation in pure Cu is approximately 0.74T_m_, which is similar to Pamato *et al*.’s FCC gold^26^. However, the observable onset temperature of vacancy formation in CoCrFeNi and CoCrFeMnNi is approximately 0.6T_m_, suggesting that vacancy formation is easier in HEAs. In particular, the onset temperature of vacancy formation in CoCrFeMnNi is approximately 100 K lower than that in CoCrFeNi. This difference may result from the different diffusivities and melting points of various constituent elements. That is, there is a competition between the element diffusivity and vacancy formation. For example, while Mn has the lowest melting temperature, the atom size of Mn is the largest in the two HEAs. When the samples are heated, the diffusion directions of different elements are a function of these multiple parameters and are thus difficult to immediately discern.

The vacancy concentrations of CoCrFeNi and CoCrFeMnNi alloys were found to be 3.25 × 10^−3^ at 1,573 K and 1.8 × 10^−3^ at 1,273 K, respectively. The CoCrFeMnNi alloy was only measured up to 1,273 K because of greater volatilization of Mn. Per Bukonte *et al*.’s derivation^32^, the Gibbs free-energy change per mole in a metallic system subjected to vacancy formation is

4 $$\varDelta G=\varDelta H-T\varDelta {S}_{conf}-T\varDelta {S}_{vib}$$

where *ΔG*, *ΔH*, *ΔS*_*conf*_, and *ΔS*_*vib*_ are the Gibbs free energy change at temperature *T*, the formation enthalpy of vacancy, the configurational entropy of vacancy, and the vibrational entropy of vacancy, respectively.

To calculate the minimization point of *ΔG*, we take the derivative of *ΔG* with respect to vacancy concentration. The vacancy concentration *X*_*v*_ can be obtained through Eq. (5):

5 $${X}_{V}=\exp (\frac{\varDelta {S}_{v}}{{k}_{B}})\exp (\frac{-\varDelta H}{{k}_{B}T}).$$

In Eq. (5), *k*_B_ is the Boltzmann constant, $\varDelta H$ is the formation enthalpy, and $\varDelta {S}_{v}$ is the formation entropy of the vacancy. The reciprocal form of Eq. (5) is used for linear fitting. The formation enthalpy and entropy can be determined by linearly fitting the natural logarithm of ${X}_{V}$ as a function of *1/T*. An expression for the formation enthalpy can be obtained from the slope of the linear fit.

**Figure 3:**
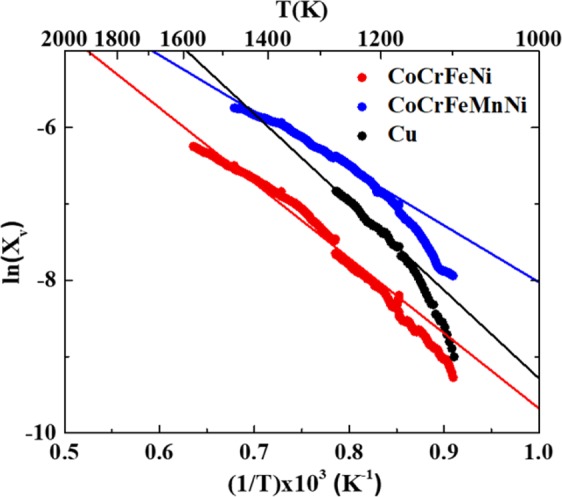
The fitted results of formation energy and entropy for vacancy formation in Cu, CoCrFeNi, and CoCrFeMnNi.

According to the Arrhenius-type assumption in Eq. (5), the fitted curves of ln(*X*_*v*_) versus *1/T* of Cu, CoCrFeNi, and CoCrFeMnNi are illustrated in Fig. 3. The formation enthalpy of Cu was calculated to be 1.00 eV, which is close to Simmons and Balluffi’s results^31^. Following the same method, we found that the vacancy formation enthalpies of CoCrFeNi and CoCrFeMnNi are 0.85 eV and 0.64 eV, respectively. Both the formation enthalpy and entropy in HEAs are lower than those in pure metals, demonstrating the easier vacancy formation in HEAs. For FCC metals, such as Ag, Au, Cu, and Al, vacancy concentrations below the melting point are generally smaller than ~9 × 10^−4 ^^21,31^. The formation enthalpies of Ag, Au, Cu, and Al are 0.78–1.24 eV, 0.6–0.962 eV, 1.191–1.33 eV, and 0.66–0.85 eV, respectively^33^. The different behaviors of the HEAs and aforementioned pure FCC metals may originate from several effects, including heterogeneous elements^34^, various bonds^35^, lattice mismatch^36^, and a manufacturing-dependent microstructure^37^. Nevertheless, we conclude that the atomic diffusion mechanism in HEAs is the vacancy mechanism in FCC metals.

To further discuss the formation entropy of vacancies in CoCrFeMnNi, it is necessary to investigate the characteristics of the vacancies. We followed Seeger and Banhart’s positron measurement^24^ method for the samples in Fig. 1(a–c) and determined the o-Ps lifetime (*τ*_3_). According to our results, *τ*_3_ of CoCrFeMnNi is 2.996 ± 0.051 ns, and *τ*_3_ of CoCrFeNi is 2.556 ± 0.078 ns, as listed in Table 1. By using the Tao–Eldrup equation^38–40^, we found that the cavity size radii of CoCrFeMnNi and CoCrFeNi are 3.634 ± 0.023 Å and 3.318 ± 0.044 Å, respectively. In contrast, Cu has the longest *τ*_3_ (3.158 ± 0.082 ns), corresponding to the largest cavity size radius (3.742 ± 0.036 Å). Furthermore, the fraction of effective free volume (*ffv*) can be semi-empirically determined as *ffv* = *CV*_*f*_*I*_3_, where *V*_*f*_ is the fraction of free volume calculated from *τ*_3_ with a spherical approximation, *I*_*3*_ is the intensity (in %) of the third mean lifetime, and *С* is an empirical parameter, which can be determined by calibrating with other physical parameters. The *ffv* of CoCrFeMnNi (0.275% ± 0.020%) and CoCrFeNi (0.225% ± 0.026%) is larger than that of Cu (0.208% ± 0.035%), indicating that cavities clustering in HEAs effectively occupy more space, forming more free volumes. The cavity size and fraction of free volume are larger in CoCrFeMnNi than in CoCrFeNi, suggesting that Mn lowers the formation energy of vacancy and exhibits element-dependent interaction^19,41^. The results also support the lower enthalpy of vacancy formation in HEAs. Because of different melting temperatures and principal element sizes, the vacancy formation is strongly governed by element-dependent behaviors rather than by high entropy effects, especially at high temperatures.

**Table 1:** Positron annihilation spectra results for the nano-cavities of Cu, CoCrFeNi, and CoCrFeMnNi.

Sample	*τ*_*3*_ (ns)	I_3_ (%)	R (Å)	ffv (%)
CoCrFeMnNi	2.996 ± 0.051	0.761 ± 0.065	3.634 ± 0.023	0.275 ± 0.020
CoCrFeNi	2.556 ± 0.078	0.818 ± 0.071	3.318 ± 0.044	0.225 ± 0.026
Cu	3.158 ± 0.082	0.527 ± 0.074	3.742 ± 0.036	0.208 ± 0.035

### Effect of Mn on HEAs

**Figure 4:**
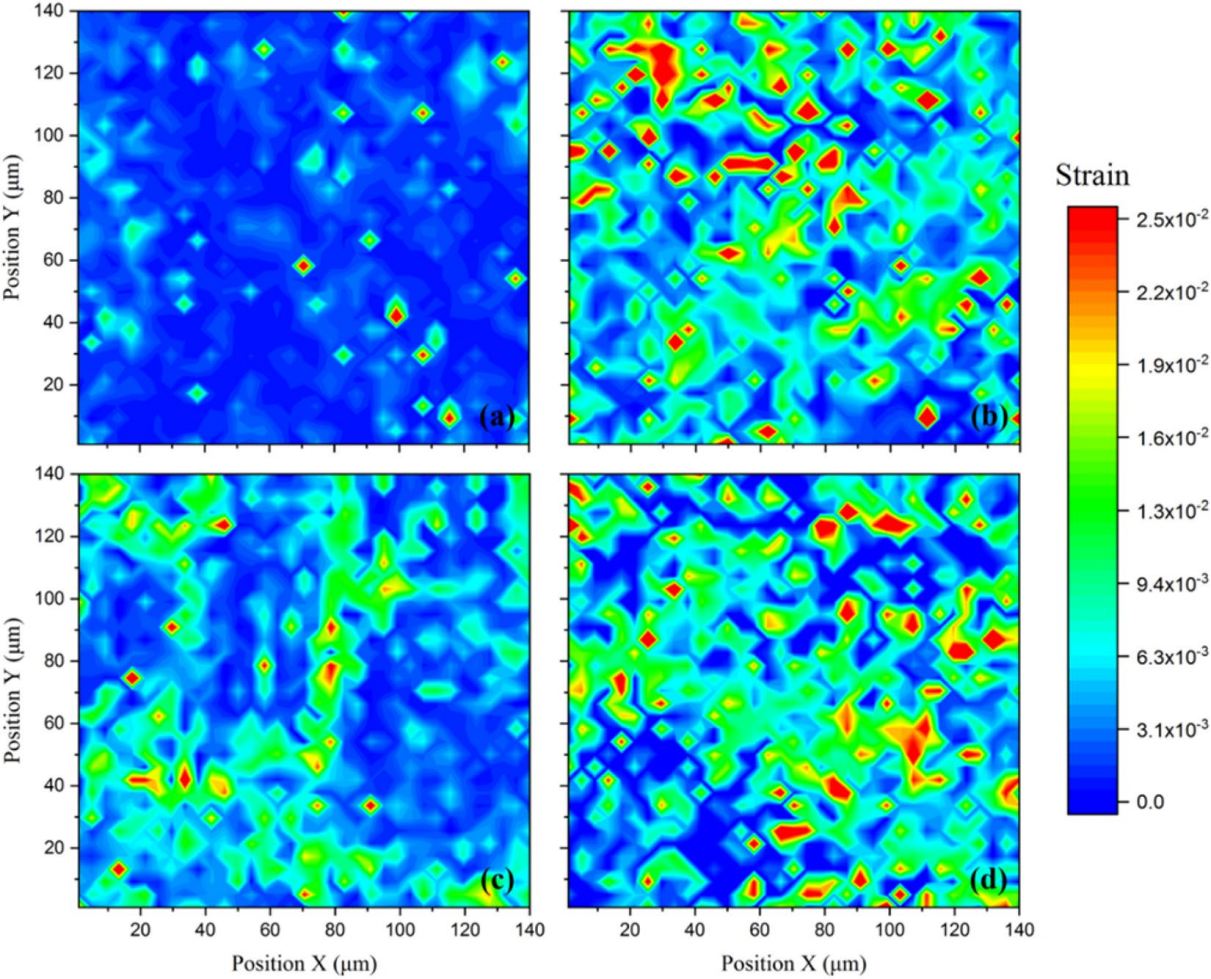
Lattice strain maps of the main diffraction peak (220) in (**a**) CoCrFeNi and (**b**) CoCrFeMnNi under non-equilibrium conditions, and the main diffraction peak (200) in (**c**) CoCrFeNi and (**d**) CoCrFeMnNi under quasi-equilibrium conditions.

Our earlier work^30^ has reported that the CoCrFeMnNi has significant creep under stress at 1,000 K. Although there is no conclusive evidence demonstrating if thermal expansion is influenced by dislocations or grain boundaries and surfaces in HEAs at higher temperatures, the residual lattice strains in the CoCrFeNi and CoCrFeMnNi alloys are quite different. These can be measured by scanning X-ray Laue diffraction. Nano-projection X-ray microscopy (PXM) measurements using high-energy X-rays facilitates the investigation beneath the surface of bulk samples of individual grains embedded in a polycrystal with a submicron spatial resolution. Figure S1 describes the Laue diffraction patterns and fitting results with the Miller indices (*hkl*) of CoCrFeNi crystals at both non-equilibrium and equilibrium states. Both CoCrFeNi and CoCrFeMnNi HEAs exhibit a main diffraction peak of (220) in the non-equilibrium state and a main diffraction peak of (200) under equilibrium conditions. Figure 4 depicts the lattice strain mapping of (220) under non-equilibrium and (200) at equilibrium conditions in the two HEAs. More uniform lattice strains are seen in CoCrFeNi (Fig. 4(a)) than in CoCrFeMnNi (Fig. 4(b)) under non-equilibrium conditions. A similar observation is apparent for the equilibrium condition, suggesting greater lattice strain heterogeneity resulting from the effect of Mn under both heating conditions. In addition, the quasi-equilibrium heating induces heterogeneous lattice strain in which the local residual lattice strains for both HEAs subjected to a quasi-equilibrium in Fig. 4(c,d) are more heterogeneous than those subjected to a non-equilibrium state in Fig. 4(a,b).

Heating induces an increasingly complex lattice structure in the CoCrFeNi and CoCrFeMoNi HEAs, which is also observable through the mapped images of crystal orientation and peak width in Figs S2 and S3, respectively. The greater lattice strain deviation caused by additional vacancy-induced distortion^42^ is found in samples subjected to a quasi-equilibrium condition, but not under non-equilibrium condition. In Figures S2 and S3, the local lattices orient differently and exhibit differently distributed defects, which can influence the geometry factor (*g*_*j*_) in Eq. (6) (shown in the discussion of divacancy). Therefore, Simmons and Balluffi’s model^27^ for various types of atomic defects, geometry factors, and defect paths in HEAs cannot be easily simplified. Magomedov^43^ proposed that the negative formation entropy derives from the realignment of a distorted crystal structure relaxed by vacancy formation. Several metallic systems such as metallic glass^44^, as well as Au–Ni^45^, and Cu–Ni^46^ systems, also exhibit negative formation-entropy effects. For HEAs^19,41,47–49^, Schön *et al*. investigated the entropy hypothesis and concluded that the competition between conflicting interactions in the solid solution seems to be the relevant factor in HEAs^41^.

The cavity size of CoCrFeMnNi is approximately 3.634 ± 0.023 Å. Comparison of the atomic radii of Co (1.25 Å), Ni (1.25 Å), Fe (1.27 Å), Cr (1.28 Å), Cu (1.28 Å), and Mn (1.35 Å)^50^ suggest that Mn has the largest atomic diameter and tends to cause a compressive stress center in its surrounding area. However, the presence of Mn locally increases the tensile strain in the remaining 80% of the other equimolar atoms, such as Co, Cr, Fe, Ni. This is evidenced by the greater heterogeneity in the lattice strain mapping results in Fig. 4(b). Indeed, the lattice parameters of CoCrFeNi and CoCrFeMnNi are 3.57 Å and 3.59 Å, respectively. It is known that the tensile strain reduces the vacancy formation enthalpy because bond breaking to form a vacancy requires less energy. Thus, the localized structural relaxation is accompanied by easier vacancy formation. The vacancy and its local shell relax neighboring atoms around the vacancy as a cluster. Pickering *et al*.’s conclusions suggest that the CoCrFeMnNi cannot be regarded as a thermodynamically stable single phase at all temperatures^51^.

### Surface segregation on HEAs

**Figure 5:**
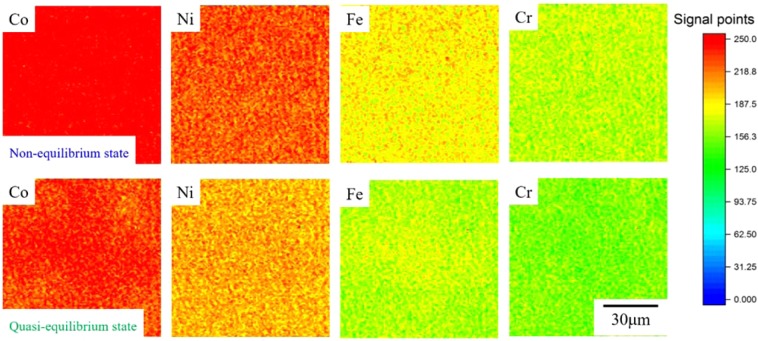
XRF maps of the CoCrFeNi HEA after non-equilibrium (upper) and quasi-equilibrium (bottom) heating.

**Figure 6:**
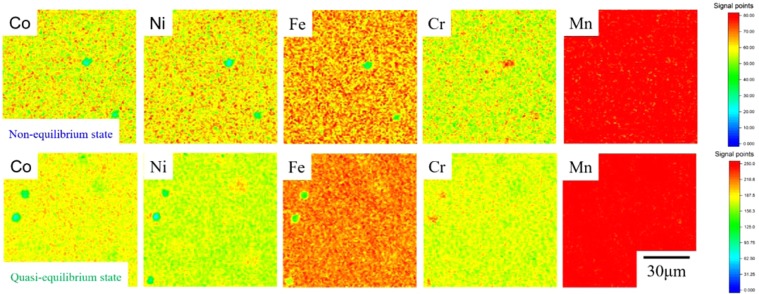
XRF maps of the CoCrFeMnNi HEA after non-equilibrium (upper) and quasi-equilibrium (bottom) heating.

Since heating activates diffusion, investigating local chemical heterogeneity induced by segregation is necessary. For pure metals, there is no surface segregation upon heating. For HEAs, we conducted an X-ray nanoprobe (XNP) study to verify the existence of surface segregation of certain elements upon heating. The XRF maps describe the element distributions for non-equilibrium and quasi-equilibrium heating of CoCrFeNi in Fig. 5 and for CoCrFeMnNi in Fig. 6. For CoCrFeNi (Fig. 5), the constituent elements Ni, Fe, and Cr demonstrate near uniformity within the scanning area under both non-equilibrium and quasi-equilibrium states. In contrast, Co has obvious non-uniformities under the quasi-equilibrium condition as compared with the non-equilibrium condition. The area of non-uniformity of the Co can be as large as 30 μm. The spatially heterogeneous Co can affect vacancy more than the other elements during quasi-equilibrium heating of CoCrFeNi. However, in Fig. 6, local Cr enrichment and depletion in the other alloying elements can be observed for both non-equilibrium and quasi-equilibrium heating of the CoCrFeMnNi alloy. Mn clusters are also found on the surface of the specimen in the non-equilibrium state; however, they disappear under quasi-equilibrium heating. Formation of Cr clusters on the surface after quasi-equilibrium heating suggests a substitution of Mn for Cr under quasi-equilibrium heating. It is expected that the different atomic radii of Mn and Cr create heterogeneous vacancy sizing.

Several studies have reported similar phenomena of Cr segregation upon heating of CoCrFeMnNi HEA. Specifically, He *et al*. reported that Cr-rich grain-boundaries precipitate after creep at temperatures ranging from 1023 to 1123 K^52^, while Schuh *et al*. detected a NiMn phase, Cr-rich phase, and FeCo phase^53^. Pickering *et al*. also indicated Cr-rich M_23_C_6_ and the σ-phase precipitation from CoCrFeMnNi subjected to prolonged heating at 700 °C^51^. Although the reported phases are not exactly the same, they may depend on the ambient conditions. If the alloy is in an oxygenated ambient environment, then we may expect the segregation of Cr to the surface since the oxidation of Cr is the most stable among the constituent elements in HEAs. In an ultrahigh vacuum, we might expect Mn to segregate to the surface as it has a high partial pressure. Thus, the surface concentration and the bulk concentration in different HEAs vary, *i.e*., CoCrFeMnNi exhibits more segregation than CoCrFeNi does. This is a unique property of HEAs. Clearly, the species and amount of surface segregation are affected by element diffusivity and surface energy changes. Moreover, because of local element heterogeneity induced by segregation, the vacancies are not exclusively single monovacancies, which are discussed as follows.

### Divacancy

The pre-exponential factor of diffusivity is dominated by entropy. Recalling Simmons and Balluffi’s model of the expected equilibrium atomic fraction of the *j*^*th*^ type of atomic defect as

6 $$c={g}_{j}exp(\frac{-{G}_{j}^{f}}{kT})={g}_{j}exp(\frac{-{S}_{j}^{f}}{T})exp(\frac{-{H}_{j}^{f}}{kT})$$

where ${G}_{j}^{f}$, ${S}_{j}^{f}$, and ${H}_{j}^{f}$ are the free energy of formation, the entropy of formation (both exclusively configurational entropies), and the energy of formation, respectively. ${g}_{j}$ is a constant geometry factor depending upon the number of possible defect orientations in the lattice^27^. If different types of defects may be present, the vacancy concentration can be quantified as

7 $${X}_{V}=({c}_{v1}+2{c}_{v2}+3{c}_{v3}+\cdots )-({c}_{i1}+2{c}_{i2}+\cdots )$$

where ${c}_{v1}$, ${c}_{v2}$, and ${c}_{v3}$ are the fractional concentrations of monovacancies, divacancies, and trivacancies, respectively, and ${c}_{i1}$and ${c}_{i2}$ are the corresponding concentrations of interstitial-type defects^27^.

Divacancy is a non-equilibrium defect in a crystal lattice where two adjacent atoms are missing^27,54,55^. In principle, divacancy formation should decrease entropy. Since there are size differences among the vacancies and different elements in HEAs, divacancy may form in alloys subjected to heating. Bukonte *et al*.’s thermodynamics derivations^32^ focus on the effect of divacancy. In their derivations^32^, the total entropy term *ΔS* consists of the vibrational entropy *ΔS*_*vib*_ and the configurational entropy *ΔS*_*conf*_. The vibrational (or thermal) entropy describes random vibrational motion in a defective crystal. Since the lattice atoms around the monovacancy and divacancy are less bound, each vacancy contributes little to the total vibrational entropy. Additionally, the space left after Mn evaporation may accommodate monovacancies or even divacancies for the other smaller atoms. Hence, at higher temperatures, when the entropy term *−TΔS*_*vib*_ in Eq. (4) is enhanced, the defect entropy due to divacancy with a variation of elements can be much more influential in HEAs than in pure metals.

Divacancy contribution at the melting point may be less than approximately 15% in pure Al^27^. In most pure metals, the interstitial-type defects can be ignored. However, the validity of the conventional assumptions of vacancy in HEAs needs further investigation. For example, Elsayed *et al*.^18^ show the majority of the vacancies from the surface but not from underneath the surface. Recalling Eq. (2), does thermal expansion due to the presence of lattice defects between the lattice and bulk specimen elongate equally? Local lattices adjacent to different defects, such as grain boundaries and dislocations, may alter the lattice frequency distribution and thus the internal energy in HEAs. Secondly, the question persists as to whether the volume changes due to the generation or destruction of atomic sites can be assumed isotopic at dislocation sources and sinks. HEAs can also create or destroy vacancies at the grain boundaries or at the surface. However, Christofidou *et al*.’s results^25^ indicate vastly different stoichiometry of phase compositions at the grain boundaries for different CoCrFeNiMn_x_ systems subjected to heating. The vacancy distributions in HEAs may not be isotropic and homogeneous as in pure metals.

As compared with pure Cu and CoCrFeNi, the Mn effect is significant for CoCrFeMnNi. If the thermal expansion of HEAs due to the presence of lattice defects is equal at the scale of the lattice and macroscopically, then there may be negative formation entropy of CoCrFeMnNi. The negative formation entropy of the CoCrFeMnNi may originate from the additional vibrational entropy due to divacancy and lattice relaxation on top of the configurational entropy at high temperatures. Alternatively, if the thermal expansion of HEAs due to the presence of lattice defects is not equal at the lattice and macroscopic levels, the vacancy effect becomes more complicated and we cannot simply estimate formation entropy based on Arrhenius-type model fitting.

## Summary

We demonstrate different chemical and lattice heterogeneous distributions in the CoCrFeNi and CoCrFeMnNi HEAs subjected to heating. The two HEAs reveal larger free volume fractions than Cu for accommodating vacancies. There is a structural rearrangement accompanying vacancy formation at elevated temperatures. The larger effective vacancy size and the tensile strain induced by the presence of Mn result in a smaller vacancy formation enthalpy in the CoCrFeMnNi than in the CoCrFeNi. Our results suggest element-dependent vacancy formation behavior in HEAs.

## Materials and Methods

### Sample preparation

The CoCrFeNi and CoCrFeMnNi HEAs were fabricated by vacuum arc melting with equal molar compositions of elemental powders with purities higher than 99.9% (in percent by weight). Before the start of the process, the base pressure was less than 8 × 10^−2^ Torr. The ingots of HEAs were then fabricated under an argon protective atmosphere of 380 Torr. To ensure chemical homogeneity, these ingots were re-melted at least three times and then cooled in a Cu mold at a controlled cooling rate. Post heat-treatment for homogenizing was performed at 1,473 K for 48 hr.

Phase identification and lattice constant measurements were obtained by a Bruker D8 Discover X-ray Diffraction System with Cu K_α_ radiation, by synchrotron X-ray diffraction measurement at the National Synchrotron Radiation Research Center (NSRRC) at beamline BL01C, and by a Vulcan neutron diffractometer at Oak Ridge National Laboratory (ORNL). Both CoCrFeNi and CoCrFeMnNi alloys exhibit a single FCC phase, with lattice constants of 3.57 Å and 3.59 Å, respectively. We note that the addition of Mn expanded the lattice of CoCrFeNi. Samples for alloy composition identification were first sanded by 2,500 grit SiC paper and polished down to 0.05 μm with an alumina compound. By scanning electron microscopy (SEM) using a JEOL 6500 with an energy-dispersive spectrometer (EDS), the constituent elements were found to be equal in both CoCrFeNi and CoCrFeMnNi alloys. The standard deviation of the alloy composition distribution was 0.5–1.2 at.% for each element.

### Measurement of vacancy concentration

For vacancy concentration measurement, Cu, CoCrFeNi, and CoCrFeMnNi cylindrical samples (6 mm in diameter and height) were prepared for thermal expansion tests using a Netzsch dilatometer (DIL402) with an accuracy in strain measurements up to 0.002%. To obtain vacancy concentrations under quasi-equilibrium conditions (Seeger’s approach), the heating rate of Cu, CoCrFeNi, and CoCrFeMnNi samples was lowered to a minimum of 0.05 K/sec. For comparison, the heating rate of the samples for non-equilibrium measurements was at 0.417 K/sec. The samples were heated at the same environmental temperature as the holding stage for quasi-equilibrium conditions.

### Positron lifetime measurements

Cu, CoCrFeNi, and CoCrFeMnNi samples were tested with a variable mono-energy slow-positron beam (VMSPB) at room temperature. This radioisotope beam used 50 mCi of ^22^Na as a positron source. The beam was operated in the range of 0–30 keV positron incident energy, equivalent to a mean depth of 0–10 mm. According to Tao’s research^39^, a well-established semi-empirical equation was derived by fitting the measured ortho-positronium (o-Ps) lifetime in a spherical infinite potential model with a known radius of cavity size (*R*). The o-Ps lifetime is denoted as *τ*_*3*_, the third mean lifetime, which is analyzed from the experimental PALS spectra^38–40,56,57^.

### Spatially resolved X-ray measurements

Two synchrotron X-ray instruments of the Taiwan Photon Source (TPS) of the NSRRC were used. The Nano X-ray Laue diffraction at TPS 21A was used in the determination of the crystal information by indexing the Laue patterns using X-ray micro-diffraction analysis software (XMAS)^58^ and for mapping the local lattice structure as d spacing for lattice strain, peak intensity for local orientation, and peak width for defects^59^. An area of 140 × 140 µm^2^ was mapped with a step size of 4.0 µm. Each mapping image contains 35 × 35 pixels, and each pixel typifies a Laue diffraction pattern. This station has a point-to-point submicron spatial resolution of 90 nm using the differential-aperture depth profiling. Samples were prepared using standard metallographic techniques prior to characterization.

The X-ray nanoprobe (XNP) at TPS 23 with a 100 nm spatial resolution was used for the complimentary chemical distributions^60^. The energy of the incident X-rays was 12.8 keV, well above the k-edge absorption energies of the constituent elements of the specimens. The excited X-ray fluorescence was collected simultaneously by a Vortex-ME4, Hitachi silicon drift detector (SDD) with energy resolution of around 150 eV. We applied both spatially resolved measurements to investigate the lattice structure and possible diffusion of the alloys subjected to heating.

## Supplementary information

Element Effects on High-Entropy Alloy Vacancy and Heterogeneous Lattice Distortion Subjected to Quasi-equilibrium Heating

## Data Availability

The data will be made available on request.
