# Supplementary material for: Element Effects on High-Entropy Alloy Vacancy and Heterogeneous Lattice Distortion Subjected to Quasi-equilibrium Heating
Source: Sci Rep. 2019 Oct 15;9:14788. doi: 10.1038/s41598-019-51297-4 (PMC6794270; doi:10.1038/s41598-019-51297-4)
Supplement: Supplementary file 1 — Element Effects on High-Entropy Alloy Vacancy and Heterogeneous Lattice Distortion Subjected to Quasi-equilibrium Heating [file 41598_2019_51297_MOESM1_ESM.pdf]

Element Effects on High-Entropy Alloy Vacancy and Heterogeneous Lattice Distortion Subjected to Quasi-equilibrium Heating

E-Wen Huang*, Hung-Sheng Chou, K. N. Tu, Wei-Song Hung, Tu-Ngoc Lam, Che-Wei Tsai, Ching-Yu Chiang, Bi-Hsuan Lin, An-Chou Yeh, Shan-Hsiu Chang, Yao-Jen Chang, Jun-Jie Yang, Xiao-Yun Li, Ching-Shun Ku, Ke An, Yuan-Wei Chang & Yu-Lun Jao

Affiliations

Department of Materials Science and Engineering, National Chiao Tung University, 1001 University Road, Hsinchu 30010, Taiwan

E-W. Huang, K. N. Tu, T.-N. Lam, Y.-W. Chang & Y.-L. Jao

Department of Materials and Optoelectronic Science, National Sun Yat-sen University, Taiwan

H.-S. Chou.

Department of Materials Science and Engineering, University of California, Los Angeles, United States

K. N. Tu

Graduate Institute of Applied Science and Technology, National Taiwan University of Science and Technology, Taipei 10607, Taiwan

W.-S. Hung

R&D Center for Membrane Technology, Chung Yuan University, Taoyuan 32023, Taiwan

W.-S. Hung

Department of Physics, College of Education, Can Tho University, Can Tho City, Vietnam

T.-N. Lam

Department of Materials Science and Engineering, National Tsing Hua University, Hsinchu 30013, Taiwan

C.-W. Tsai, A.-C. Yeh, S.-H. Chang, Y.-J. Chang & J.-J. Yang

National Synchrotron Radiation Research Center, Hsinchu 30076, Taiwan

C.-Y. Chiang, B.-H. Lin, X.-Y. Li & C.-S.n Ku

Spallation Neutron Source, Oak Ridge National Laboratory, Oak Ridge, TN 37831, United States

K. An

Corresponding author

Correspondence to E-Wen Huang [EwenHUANG@nctu.edu.tw]

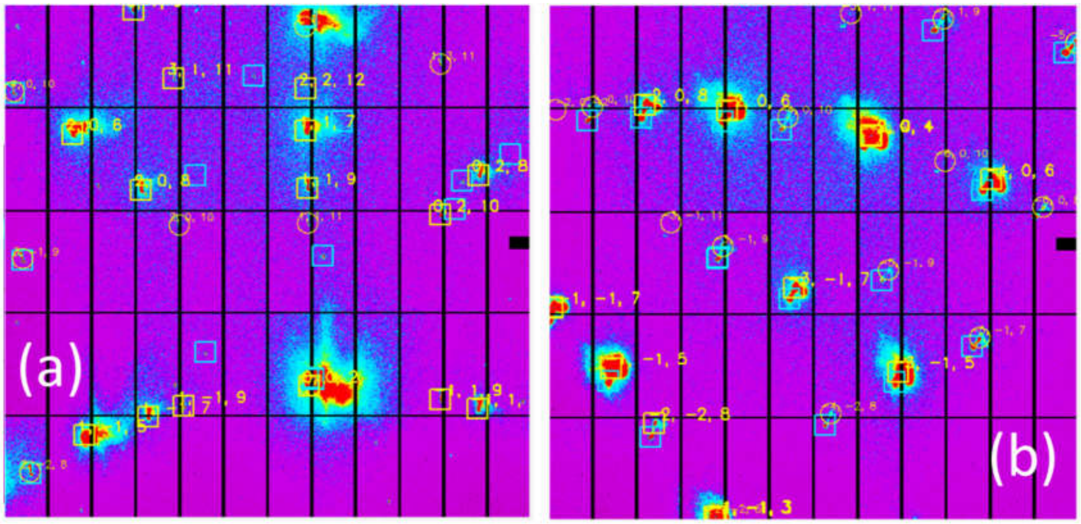

Fig. S1. The Laue diffraction patterns and fitted results with the Miller indices (*hkl*) of CoCrFeNi crystals at the (a) quasi-equilibrium and (b) non-equilibrium states.

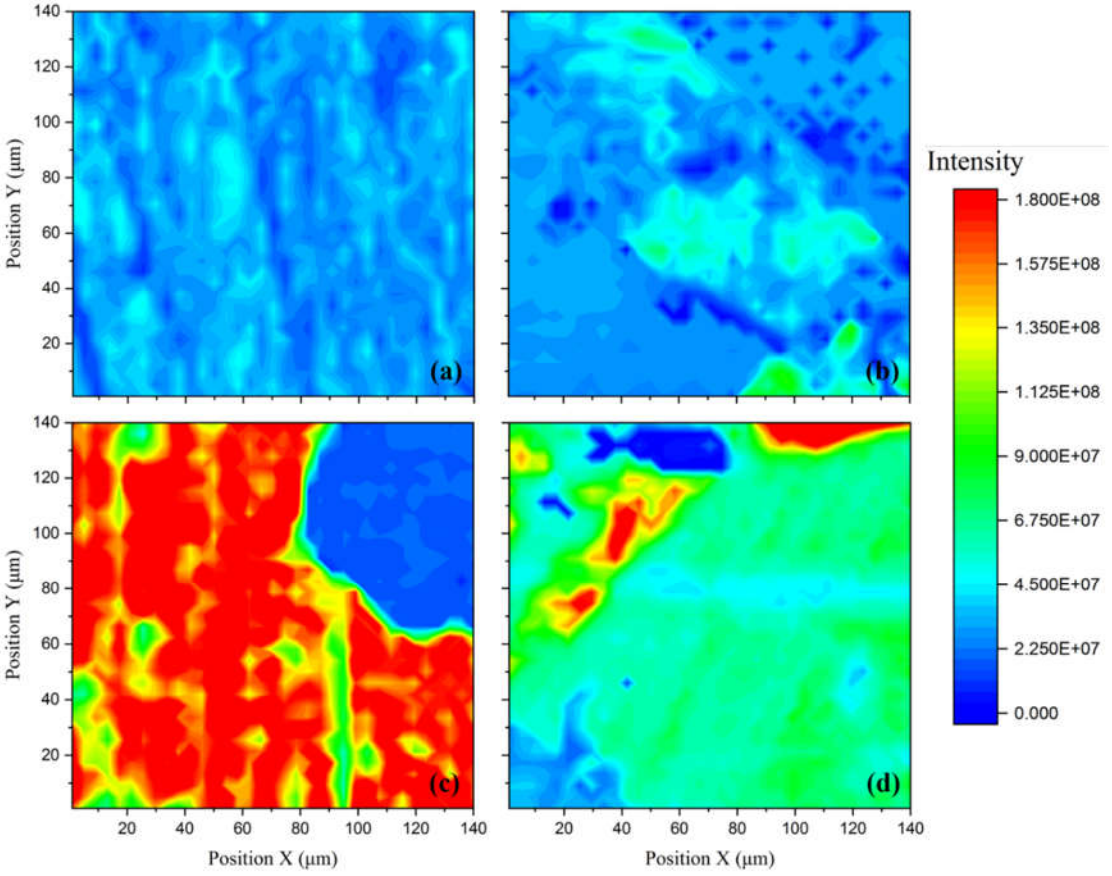

Fig. S2. Crystal orientation maps of the main diffraction peak (220) in (a) CoCrFeNi and (b) CoCrFeMnNi under non-equilibrium conditions, and the main diffraction peak (200) in (c) CoCrFeNi and (d) CoCrFeMnNi under quasi-equilibrium conditions.

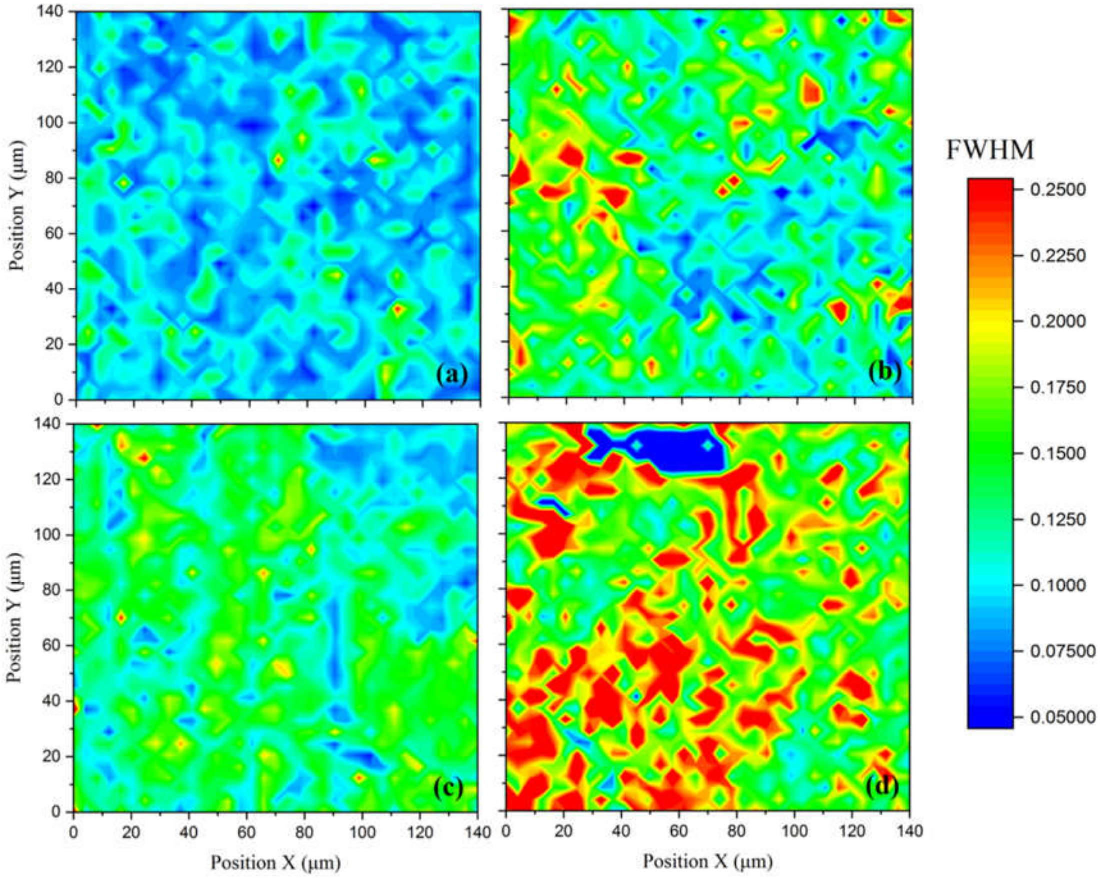

Fig. S3. Full width maps of the main diffraction peak (220) for (a) CoCrFeNi and (b) CoCrFeMnNi under non-equilibrium conditions, and the main diffraction peak (200) in (c) CoCrFeNi and (d) CoCrFeMnNi under quasi-equilibrium conditions.

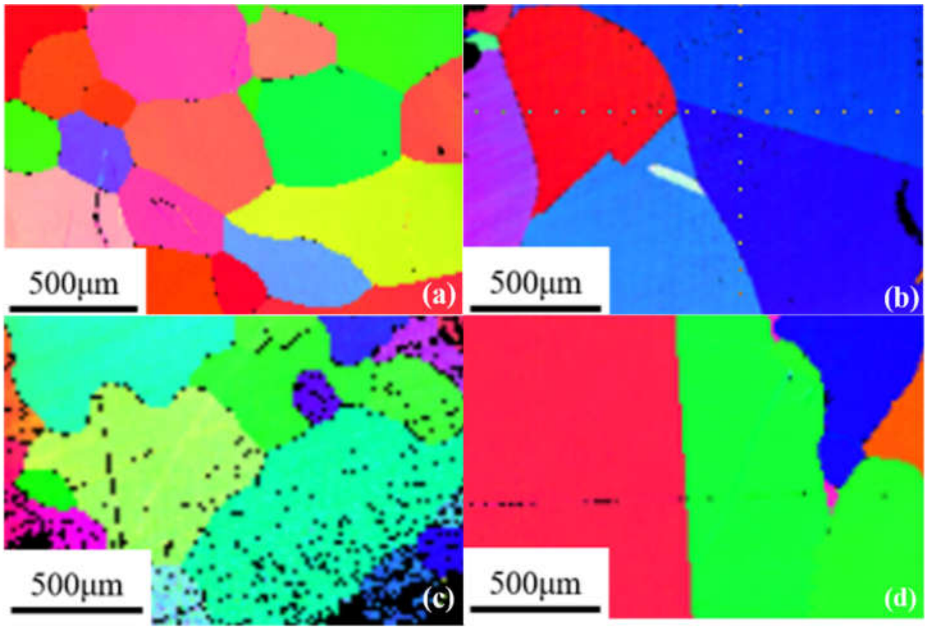

Fig. S4. EBSD analysis in (a) CoCrFeNi and (b) CoCrFeMnNi under non-equilibrium conditions, in (c) CoCrFeNi and (d) CoCrFeMnNi under quasi-equilibrium conditions.
